# HIF3A Inhibition Triggers Browning of White Adipocytes *via* Metabolic Rewiring

**DOI:** 10.3389/fcell.2021.740203

**Published:** 2022-01-12

**Authors:** Francesca Cuomo, Carmela Dell’Aversana, Teresa Chioccarelli, Veronica Porreca, Francesco Manfrevola, Chiara Papulino, Vincenzo Carafa, Rosaria Benedetti, Lucia Altucci, Gilda Cobellis, Gilda Cobellis

**Affiliations:** ^1^ Department of Precision Medicine, Università degli Studi della Campania “Luigi Vanvitelli”, Napoli, Italy; ^2^ Institute Experimental Endocrinology and Oncology “Gaetano Salvatore” (IEOS)–National Research Council (CNR), Napoli, Italy; ^3^ Department of Experimental Medicine, Università degli Studi della Campania “Luigi Vanvitelli”, Napoli, Italy; ^4^ Biogem Institute of Molecular and Genetic Biology, Ariano Irpino, Italy

**Keywords:** browning, adipose tissue, HIF3α, thermogenesis, sirts; CB1

## Abstract

Maintenance of energy balance between intake and expenditure is a prerequisite of human health, disrupted in severe metabolic diseases, such as obesity and type 2 diabetes (T2D), mainly due to accumulation of white adipose tissue (WAT). WAT undergoes a morphological and energetic remodelling toward brown adipose tissue (BAT) and the BAT activation has anti-obesity potential. The mechanisms or the regulatory factors able to activate BAT thermogenesis have been only partially deciphered. Identifying novel regulators of BAT induction is a question of great importance for fighting obesity and T2D. Here, we evaluated the role of *Hif3α* in murine pre-adipocyte 3T3-L1 cell line, a versatile and well characterized biological model of adipogenesis, by gain- and loss-of function approaches and in thermogenesis-induced model *in vivo*. *HIF3A* is regulated by inflammation, it modulates lypolysis in adipose tissue of obese adults, but its role in energy metabolism has not previously been investigated. We characterized gene and protein expression patterns of adipogenesis and metabolic activity *in vitro* and mechanistically *in vivo*. Overexpression of *Hif3α* in differentiating adipocytes increases white fat cells, whereas silencing of *Hif3α* promotes “browning” of white cells, activating thermogenesis through upregulation of *Ucp1, Elovl3, Prdm16, Dio2* and *Ppargc1a* genes. Investigating cell metabolism, Seahorse Real-Time Cell Metabolism Analysis showed that silencing of *Hif3α* resulted in a significant increase of mitochondrial uncoupling with a concomitant increase in acetyl-CoA metabolism and Sirt1 and Sirt3 expression. The causal *Hif3α/Ucp1* inverse relation has been validated in Cannabinoid receptor 1 (CB1) knockout, a thermogenesis-induced model *in vivo*. Our data indicate that *Hif3α* inhibition triggers “browning” of white adipocytes activating the beneficial thermogenesis rewiring energy metabolism *in vitro* and *in vivo*. *HIF3A* is a novel player that controls the energy metabolism with potential applications in developing therapy to fight metabolic disorders, as obesity, T2D and ultimately cancer.

## Introduction

The frequency of metabolic disorders, such as obesity and type 2 diabetes (T2D) rose up over the last 50 years and the generation of novel therapeutic approaches aimed to ameliorate the balance of energy metabolism in adipose tissue is going to be developed (Tsalamandris et al., 2019). The adipose tissue is a metabolically dynamic organ, classified as white (WAT) and brown (BAT) adipose tissues with distinctive features. WAT can store energy as triglycerides in response to caloric oversupply, whereas BAT produces heat in response to cold and/or hormonal stimulation, a process called non-shivering thermogenesis. Morphologically, WAT cells are larger, spherical with unilocular lipid droplets helpful for lipid storage. BAT cells are smaller with numerous, little lipid droplets, helpful for lypolysis. Obesity is linked to excessive WAT accumulation, chronic low-grade inflammation, energy imbalance and altered metabolism, prompting the development of insulin resistance, dyslipidaemia behind the high body mass index (BMI) (Townsend and Tseng, 2012). In contrast, BAT cells are linked with low BMI, low fat mass, normal glucose and cholesterol plasma levels. WAT cells can adopt a thermogenic phenotype in response to environmental stimuli and dietary lifestyle, representing a promising strategy to counteract the metabolic disorders. However, the causal factors that promote the BAT emergence have been only partially deciphered.

The hypoxia-inducible transcription factors (*HIF1A* and *HIF2A*) are involved in metabolic changes driving cell adaptation to low oxygen availability (Semenza, 2016). Few are known on *HIF3A* function in humans. Previous reports revealed that, in humans, *HIF3A* locus is significantly hypermethylated in adipose tissue of obese adults through genome-wide methylome analyses (Dick et al., 2014), modulates lypolysis (Kulyte et al., 2020), correlated with increased risk of insulin resistance and glucose metabolism (Haertle et al., 2017) and, in murine pre-adipocyte 3T3-L1 cells, *Hif3α* regulates WAT adipogenesis (Hatanaka et al., 2009). We recently demonstrated that *HIF3A* is induced by pro-inflammatory cytokines in an oxygen-independent manner in mesenchymal stem cells (MSCs), suggesting that it was involved in sensing inflammation rather than hypoxic stress (Cuomo et al., 2018). Then, we hypothesized that *HIF3A* might connect cell metabolism to energy demand through appropriate gene expression regulation. In this study, we investigated the expression of *Hif3α* in the pre-adipocyte 3T3-L1-cell line, which is a versatile and well-characterized biological model of adipogenesis. Raising *Hif3α* levels by pro-inflammatory cytokines and/or forced *HIF3A* expression induces WAT accumulation, whereas *Hif3α* knockdown induces the appearance of brown/beige adipocytes, increases mitochondrial uncoupling via *Ucp1*, linked to acetyl-CoA and Sirt1 and Sirt3 accumulation. To validate our results, the cannabinoid receptor 1 knockout (CB1KO) mouse, an established thermogenesis-induced model (Wagner et al., 2011), has been exploited, showing the appearance of “browning” of white fat via *Hif3α*/Ucp1 inverse relation.

These findings suggest that *Hif3α* is a regulator of BAT induction, guiding metabolic reprogramming, opening new perspective in obesity treatment, also as a complementary approach to cancer therapy.

## Materials and Methods

### 3T3-L1 Culture

Grown and differentiation of 3T3-L1 cells were previously described (Zebisch et al., 2012). Briefly, cells were grown in Dulbecco’s modified Eagle’s medium (DMEM) (Euroclone SPA, Italy), containing 10% heat-inactivated foetal bovine serum (FBS), 1% Penicillin-streptomycin, and 2 mM L-Glutamine in a humidified atmosphere containing 5% CO_2_ at 37°C. Induction of adipose differentiation was obtained by treatment with a mixture of insulin (10 ug/ml), dexamethasone (1uM) and 3-isobutyl-1-methylxanthine (IBMX, 0.5 mM) on post-confluent cells for 3 days, then changed to DMEM plus insulin (10 ug/ml). Different cytokines were added on post-confluent cells, according to experimental design (EGF 5 ng/ml, MCP1 and 20 ng/ml, respectively, Provitro, Berlin, Germany, VEGF 25 ng/ml, TNFα 10 ng/ml, IL6 20 ng/ml, IFNγ 100 ng/ml ISOkine, ORF genetics, Kopavogur, Iceland).

### Quantification of Lipids With Oil Red O Staining

Lipid accumulation was visualized by light microscopy in immature and mature adipocytes plated in 12-well plates at differentiation days 3, 7 and 10. Cells were fixed with 4% formalin in PBS for 1 h and rinsed with distilled water at RT. The fixed cells were treated with 60% isopropyl alcohol for 5 min and then stained with 0.3% Oil Red O in 60% isopropyl alcohol solution for 15 min at RT and subsequently washed with distilled water. Accumulation of lipids was quantified by isopropanol extraction for 15 min and absorbance measured with Infinite M1000 microplate reader (TECAN) at 540 nm.

### Overexpression and Silencing Experiments

3T3-L1 cells were seeded into 6-well plates at 3 × 10^5^ cells/well density. After 24 h, cells were transfected with 50 nM of small interfering RNAs (siRNAs) targeting *Hif3α* (ID 64344, Riboxx, Life Sciences, DE) or non-targeting siRNA pool for silencing experiments. Overexpression experiments were conducted using the pM02 plasmid containing the ORF of HIF3A (Z1165) purchased by GeneCopoeia with a CMV promoter. Both experiments were performed using Lipofectamine 2000 (Life Technologies, United States), according to the manufacturer’s protocol. The day after transfection, cytokines were added in the differentiation medium and cells harvested at differentiation days 3 and 7.

### RNA Preparation and qRT-PCR

Total RNA was isolated from mouse FAT and from 3T3-L1 cells using TRIzol Reagent (Invitrogen Life Technologies, Paisley, United Kingdom) following the manufacturer’s instructions. In brief, the mouse samples were homogenized in TRIzol Reagent (1 ml/100 mg of tissue); then samples (mouse and cells) were incubated for 5 min at 20°C for the dissociation of nucleoprotein complexes and added 0.2 ml chloroform/ml TRIzol Reagent following centrifugation at 12000xg for 15 min at 4°C. The aqueous phase containing the total RNA was precipitated by mixing isopropyl alcohol (0.5 ml/ml TRIzol Reagent) at −20°C O.N. After centrifugation at 120,00 x g for 10 min at 4°C, the pellet of RNA was washed with 75% ethanol, centrifuged at 7,500 x g for 10 min at 4°C and dissolved in an appropriate volume of DEPC water. The quantity and the purity of total RNAs were measured by NanoDrop 200 spectrophotometer (Thermo, Waltham, MA, United States). Then 1 ug/ml were converted to cDNA by reverse-transcription using Luna Script RT Super Mix Kit (Biolabs E3010). qRT-PCR was performed using SybrGreen PCR Master mix 2x reagent in the CFX96TM Real Time PCR Detection Systems (BioRad, CA, United States). Gene expression was normalized to the internal references’ genes (18S and β-actin). Relative expression was calculated using 2^(−ΔΔCt)^ method and exhibited as heatmap by R package.

### Protein Extraction and Western Blot Analysis

Cells were lysated in 20 mM Tris-HCl, 100 mM NaCl, 10 mM MgCl_2_, 1% NP-40, 10% glycerol, 0.1 M NaF, 100 μM Na_3_VO_4_ and protease inhibitors mixture (Roche Ltd., Basel, Switzerland). Equal amounts of proteins were separated by SDS-polyacrylamide gels and transferred to nitrocellulose membranes (Whatman, GE Healthcare, Europe). Membranes were incubated with blocking buffer (TBS-Tween buffer containing 5% milk) for 1 h at RT and subsequently with primary antibodies (see below) at 4°C ON. After three washes for 10 min with T-TBS buffer (50 mM Tris-HCl, pH 8.0, 150 mM NaCl, and 0.5% Tween-20), the membranes were incubated with horseradish peroxidase-conjugated anti-rabbit antibody (1:10,000, Amersham, GE Healthcare, Life Technologies, Europe) for 1 h at RT and then washed for 15 min with T-TBS buffer. The resulting immunocomplexes were detected using Amersham ECL Plus (GE Healthcare, Life Technologies, Europe).

### Mitochondrial Stress and ATP Production

Mitochondrial function was assessed and analysed in live cells by Seahorse XF Cell Mito Stress Test Kit (Agilent Technologies #103015), according to the manufacturer’s protocol. Briefly, 2×10^4^ cells/well of 3T3-L1 cells were plated on a Seahorse XF24 cell culture microplate and transfected with si*Hif3α*. At 24 h, the medium was replaced with differentiated medium in presence of pro-inflammatory cytokines. After 72 h, the plate was loaded into the by Seahorse XF24 Analyser (Agilent Technologies) and analysed as reported in (Carafa et al., 2020). Multiple parameters are evaluated including basal respiration, ATP-linked respiration, maximal and reserve capacities, and non-mitochondrial respiration.

### Experimental Animals and Tissue Collection

Mouse protocols were approved by the Italian Ministry of Education and the Italian Ministry of Health, with authorization n°941/2016-PR issued on 10.10.2016. Procedure involving animal care was carried out in accordance with the National Research Council’s publication *Guide for Care and Use of Laboratory Animals* (National of Institutes of Health Guide). Mice were housed in a specific pathogen-free facility, kept under controlled environmental conditions (12 h light-dark cycle, 22°C), maintained on a standard diet with free access to water. Heterozygous males and females (CD1 strand) carrying a *Cb1 null* deletion (Ledent et al., 1999) were crossed to generate CB1KO mice. Genotypes were determined by PCR using genomic DNA ad described below; body weight was measured before killing after 12 h fasting. WT and CB1KO males’ mice (8–12 months) were killed by cervical dislocation under anaesthesia, and visceral adipose tissue (vFAT) and subcutaneous inguinal white fat (iFAT) were rapidly sampled (de Jong et al., 2015; Bagchi and MacDougald, 2019) and fixed for histological analysis or frozen in liquid nitrogen before being stored at −80°C for RNA extraction.

### Genomic PCR

For genomic DNA extraction, 1–3 mg tail tip biopsy from 10-day-old pups placed into 300 µl lysis buffer (100 mM Tris-HCl, pH 8.0, 200 mM NaCl, 5 mM EDTA pH 8.0, 0.2% SDS) with 4 µl of Proteinase K (10 mg/ml in 50 mM TrisHCl, pH 8.0; Invitrogen, Carlsbad, CA, United States) and digested at 600 rpm at 56°C ON. The samples were then heated at 95°C for 15 min to inactivate proteinase K and were briefly centrifuged at 13,000×g for 1 min. After centrifugation, genomic DNA (gDNA) was isolated by the traditional phenol/chloroform method and the amount of gDNA determined by spectrophotometry (NanoDrop ND-1000 system–NanoDrop Technologies, Inc., Wilmington, DE, United States). The purity of gDNA was determined by ratio A260/A280 = 1.8.

The genotype was determined by PCR analysis of gDNA using specific primers for neomycin-cassette (mutated allele) and CB1 gene as already reported [12], at the following conditions: 94°C for 5 min, 1 cycle; 94°C for 1 min, 56°C for 1 min, 72°C for 1 min, 35 cycles; and 72°C for 5 min, 1 cycle. Finally, 25 μl of PCR amplification mixtures were analysed by electrophoresis on 1.3% agarose gel and stained with 0.5 μg/ml of ethidium bromide.

### Histological Analysis and Adipocyte Count

vFAT and iFAT mass from WT and CB1KO animals were fixed in Bouin’s solution for 12 h, dehydrated in ethanol, cleared in xylene and embedded in paraffin. Sections (13 μm) were cut and processed for haematoxylin-eosin stain and used for adipocyte count, as already reported (Suglia et al., 2016).

Stained sections were early observed under a light microscope (Leica Microsystems Inc., Milano, Italy) then density of fat mass (*i.e*., number of adipocytes per area) was analysed. Random not serial sections (*n* = 10/genotype) were used to capture the images (50× final magnification) by a high-resolution digital camera (DC300F; Leica Microsystems). The density of adipocytes was calculated per optical field. In particular, for each section a minimum of 20 random optical fields were selected and subjected to count of adipocytes. Results were expressed as mean value of number of cells per 100 mm^2^ ± SEM.

### Antibodies and Oligonucleotides

Mouse monoclonal anti Sirt1 (Abcam #110304), anti Gapdh (sc #47724, Santa Cruz Biotech) anti Tubulin (Santa Cruz Biotech #5286), and rabbit polyclonal anti LC3-I/II (abcam #41420), anti AceCs1 (cell signalling #CD19C6), anti Acc (cell signalling #C83B10), anti PDH (cell signalling #2784), anti Sirt3 (abcam #40963–100), anti Hif3α (Proteintech #27650-1-AP) anti Actin (Santa Cruz Biotech #1615) were used for western blotting. The oligonucleotides used in this study are reported in Table 1).

**TABLE 1:** Oligonucleotides used in the study.

Gene target	Sequence (5′-3′)
*mActin Fw*	ACG​TTG​ACA​TCC​GTA​AAG​ACC​T
*mActin Rw*	GCA​GTA​ATC​TCC​TTC​TGC​ATC​C
*18S Fw*	TTC​CGA​TAA​CGA​ACG​AGA​CTC​T
*18S Rw*	TGG​CTG​AAC​GCC​ACT​TGT​C
*mHif3α 1b-2 Fw*	GAG​AGG​ACA​GAG​GGC​CTT​AGG
*mHif3α 1b-2 Rw*	TGA​TTG​TGA​GGC​GCA​TGA​TG
*mHif3α 1a-2 Fw*	GGG​CGA​GCC​ATG​GCG​TTG​GG
*mHif3α 1a-2 Rw*	TAG​CTG​ATT​GTG​AGG​CGC​AT
*hHIF3A 1a Fw*	GACTGGCGAGCCATGGCG
*hHIF3A ex 2 Rw*	CAC​CTG​GAC​AAG​GCC​TCT​AT
*hHIF3A 1b Fw*	GTG​CGC​ACC​CAC​TCG​TAA​CTC​G
*hHIF3A ex 2 Rw*	CAC​CTG​GAC​AAG​GCC​TCT​AT
*hHIF3A Fw*	GGG​AAG​CTT​GCC​ATG​GCG​CTG​GGG​CTG​CA
*hHIF3A ex 2 Rw*	CAC​CTG​GAC​AAG​GCC​TCT​AT
*mPparg Fw*	TCG​CTG​ATG​CAC​TGC​CTA​TG
*mPparg Rw*	GAG​AGG​TCC​ACA​GAG​CTG​ATT
*mClec10a Fw*	AGA​CAA​CAC​CAC​CTC​CAA​GA-3′
*mClec10a Rw*	AGT​TCC​TGC​CTG​TGA​TCC​TC
*mPpargc1a Fw*	GAA​GTC​CCA​TAC​ACA​ACC​GC
*mPpargc1a Rw*	GTG​ACT​CTG​GGG​TCA​GAG​GA
*mElovl3 Fw*	CTC​ATC​GTT​GTT​GGC​CAG​AC
*mElovl3 Rw*	CCG​TGT​AGA​TGG​CAA​AGC​AC
*mUcp1 Fw*	CTC​AGG​ATT​GGC​CTC​TAC​GA
*mUcp1 Rw*	ACT​GCC​ACA​CCT​CCA​GTC​AT
*mC/ebpα Fw*	TTA​CAA​CAG​GCC​AGG​TTT​CC
*mC/ebpα Rw*	GGC​TGG​CGA​CAT​ACA​GTA​CA
*mPrdm/16 Fw*	CAG​CAA​CCT​CCA​GCG​TCA​CAT​C
*mPrdm/16 Rw*	GCG​AAG​GTC​TTG​CCA​CAG​TCA​G
*mDio2 Fw*	CTT​CCT​CCT​AGA​TGC​CTA​CAA​AC
*mDio2 Rw*	GGC​ATA​AAT​TGT​TAC​CTG​ATT​CAG​G
*mFasn Fw*	CTT​CGC​CAA​CTC​TAC​CAT​GG
*mFasn Rw*	TTC​CAC​ACC​CAT​GAG​CGA​GT
*mAngptl4 Fw*	ACT​TCA​GAT​GGA​GGC​TGG​AC
*mAngptl4Rw*	TCC​GAA​GCC​ATC​CTT​GTA​GG
*mCd36 Fw*	TCC​TCT​GAC​ATT​TGC​AGG​TCT​ATC
*mCd36 Rw*	AAA​GGC​ATT​GGC​TGG​AAG​AA
*mCb1 Fw*	CTG​ATC​CTG​GTG​GTG​TTG​AT
*mCb1 Rw*	CCT​CAG​AGC​ATA​GAT​GAT​GG

### Statistical Analysis

All tests were executed in triplicate and repeated three times. Standard error of mean (SEM) bars was reported. Student’s t-test was performed: ∗*p* < 0.05; ∗∗*p* < 0.01; and ∗∗∗*p* < 0.001.

## Results

### Modulation of *Hif3α* Expression Regulates the Adipocyte Differentiation Toward WAT Phenotype

**FIGURE 1:**
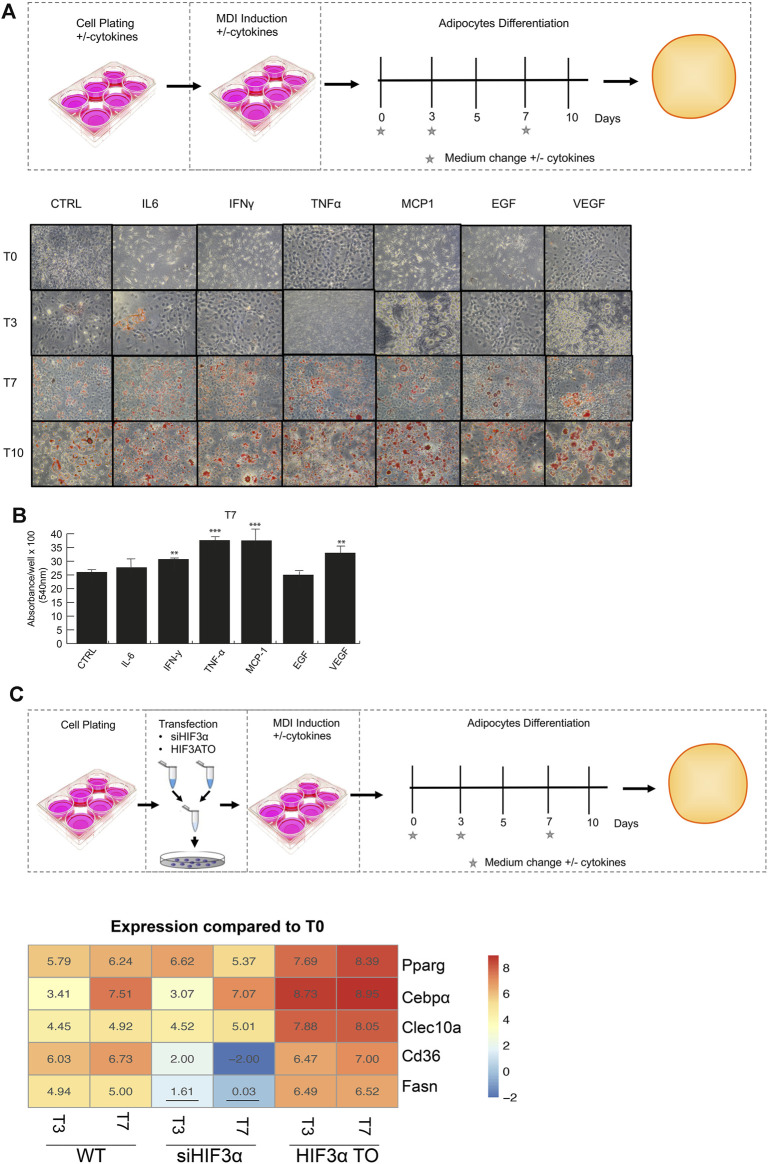
Modulation of *Hif3α* expression regulates the adipocyte differentiation toward WAT phenotype. **(A)** Schematic representation of experimental design. 3T3-L1 cells were plated in presence and absence of pro-inflammatory cytokines the day before the differentiation induction until the appearance of mature adipocytes (T0 to T10). Representative Oil Red O-staining by light microscopy in our experimental condition. **(B)** Quantification of Oil Red O-staining at 540 nm (*n* = 3) using a TECAN robotic station in mature adipocytes at day 7. ***p ≤ 0.001, **p ≤ 0.01 *vs*. control. **(C)** Schematic representation of transfection design and gene expression analysis (*Pparg, C/ebpα, Clec10a, Cd36, Fasn*) in WT, si*Hif3α* and *HIF3A*TO adipocytes. Heatmap represents expression profile of WAT genes differentially expressed during adipogenesis. Values represent the means of the fold change *vs.* T0WT (*n* = 3). T0WT is calculated as 1, thus >1 means upregulation, <1 means downregulation. All values are significant (*p* ≤ 0.01) except those underlined.

We recently showed that pro-inflammatory cytokines modulate *HIF3A* expression in an oxygen-independent manner in MSCs [8], therefore, we asked whether the cytokine-mediated effects could regulate fat metabolism and differentiation. The preadipocyte 3T3-L1 cells were exposed to pro-inflammatory cytokines (IL6, IFNγ, TNFα, MCP-1) and angiogenic markers (EGF and VEGF), which are secreted by hypercaloric state adipocytes and angiogenesis, respectively (Wernstedt Asterholm et al., 2014). After 24 h, the cells were shifted to adipocyte differentiation medium supplemented with cytokines and stained with a lipid-specific agent Oil Red O. Lipid accumulation was analysed at differentiation days 3, 7, 10 and compared to untreated cells. As shown, larger and abundant red + droplets were seen in presence of almost of cytokines in maturating adipocytes (T7 and T10) (Figure 1A), confirmed by lipid quantification (Figure 1B). To elucidate the role of *Hif3α* expression on adipocyte differentiation, two different 3T3-L1-derived cells, *i.e., HIF3A* transient overexpressing *HIF3A* (*HIF3A* TO) and *Hif3α* knocking down (si*Hif3α*) cells, were generated and the impact of *Hif3α* deregulation characterized (Supplementary Figures S1A,B). Endogenous *Hif3α* isoforms (qPCR for exon 1a: isoform 2,4,9 and 1b: isoform 7 and 8) were induced during adipocyte differentiation, in WT and TO cells, and almost all pro-inflammatory cytokines enhanced its expression (Supplementary Figures S1A,C).

In siRNA-treated (KD) cells, *Hif3α* RNA and protein were almost abolished (Supplementary Figure S1B), and its expression was not rescued by cytokines, suggesting that *Hif3α* is under the control of pro-inflammatory cytokines also in this cellular model (Supplementary Figure S1C).

To evaluate the impact on adipocyte differentiation, we monitored the expression of WAT markers; the peroxisome proliferator-activated receptor gamma (*Ppar*g), the CCAAT enhancer binding protein alpha (*c/Ebpα*) and the C-type lectin domain containing 10A (*Clec10a*) and genes involved in lipid storage or energy supply as Cd36 molecule (*Cd36*) and fatty acid synthase (*Fasn*). The experiments were performed in overexpressing (*HIF3A*-TO) and downregulating (si*Hif3α*) cells and compared to wild type cells (WT) in immature (T0) and mature adipocytes (T3 and T7) (Figure 1C). The heatmap showed the expression of WAT specific markers during adipocytes differentiation compared to WT. A significant increment of *Ppar*g*, c/Ebpα* and *Clec10a* expression was observed in differentiating WT adipocytes and in *HIF3A*-TO adipocytes, confirming that *Hif3α* expression is involved in differentiation of WAT-like adipocytes. In contrast, siRNA-mediated downregulation of *Hif3α* expression reduced the induction of WAT markers (Figure 1C). In addition, pro-inflammatory cytokines further enhanced WAT markers expression both in WT and *HIF3A*-TO cells during differentiation, confirming the link between inflammation and white adipogenesis via *HIF3A*. (Supplementary Figure S2A). To note, although TNF-α induced a reduction of stored lipids in mature adipocytes (Palacios-Ortega, S., 2015), this reduction was not still evident in our conditions (day7), since the effects are time- and dose-dependent.

### *Hif3α* Inhibition Redirects Adipocyte Differentiation Toward BAT Phenotype

**FIGURE 2:**
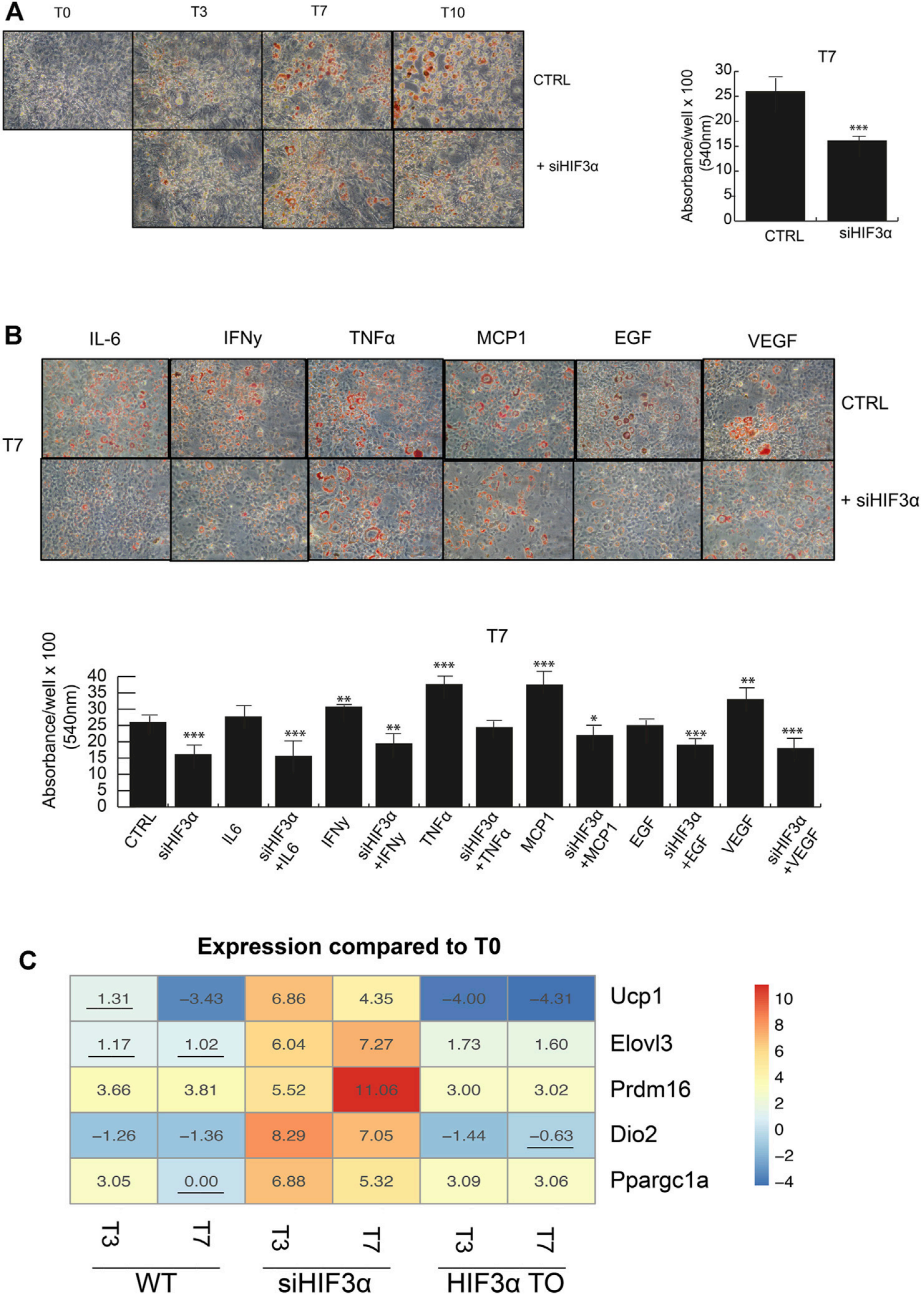
*Hif3α* inhibition redirects adipocyte differentiation toward BAT phenotype **(A,B)** 3T3-L1 cells were plated and transfected with si*Hif3α*, and exposed to pro-inflammatory cytokines and differentiation medium until the appearance of mature adipocytes (T0 to T10). Representative Oil Red O-staining by light microscopy in our experimental condition. Quantification of Oil Red O-staining at 540 nm (*n* = 3) using a TECAN robotic station in mature adipocytes at day 7. ***p ≤ 0.001, **p ≤ 0.01, *p ≤ 0.05 *vs*. control. **(C)** Analysis of BAT gene regulation (*Ucp1, Elovl3, Prdm16, Dio2, Ppargc1a*) in WT, si*Hif3α* and *HIF3A*TO adipocytes. Heatmap represents expression profile of BAT genes differentially expressed across the different experimental conditions. Values represent the means of the fold change *vs.* T0WT (*n* = 3). T0WT is calculated as 1, thus >1 means upregulation, <1 means downregulation. All values are significant (*p* ≤ 0.01) except those underlined.

Lipid droplets (LD) are not only a passive storage of lipids, but dynamic organelles that play a role in energy metabolism. We evaluated if and how the consistent downregulation of WAT markers (*Ppar*g, *c/Ebpα, Clec10a, Cd36 and Fasn*) in si*Hif3α* cells impacted the adipocyte phenotype. Cells were induced to differentiate and mature adipocytes stained with Oil Red O and quantified. A marked decrease in lipid abundance is observed in si*Hif3α* mature adipocytes, compared to the control (Figure 2A) and the cytokines pre-treatment did not rescue the LD abundance (Figure 2B). Then, we analysed the expression of specific markers of brown/beige adipocytes, as uncoupling protein 1 (*Ucp1*), elongation of very long chain fatty acids 3 protein (*Elovl3*), positive regulatory domain containing 16 (*Prdm16*), type II iodothyronine deiodinase (*Dio2*) and peroxisome proliferator-activated receptor gamma coactivator 1-alpha (*Ppargc1a*). Heatmap showed that the expression of these markers was massively increased in si*Hif3α*-derived adipocytes, suggesting a transition toward “brown/beige” phenotype (Figure 2C). In TO adipocytes, these markers were barely detectable. Therefore, silencing of *Hif3α* mediates the appearance of BAT-like adipocytes via *Ucp1* upregulation and a consistent downregulation of WAT markers.

### Hif3α Silencing Enhances Lipid Catabolism

**FIGURE 3:**
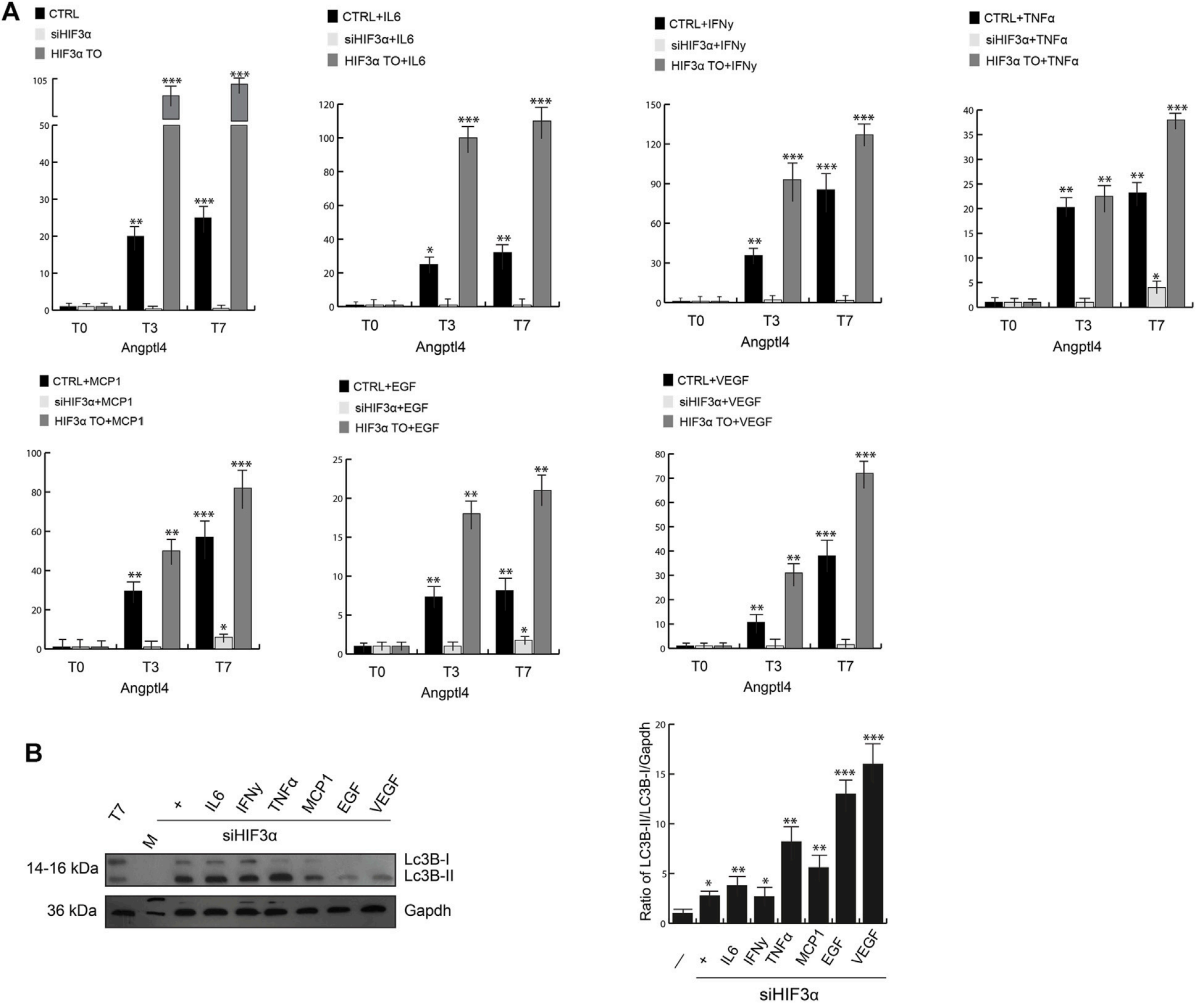
*Hif3α* silencing enhances lipid catabolism. **(A)** Expression levels of *Angptl4* by qPCR in WT, si*Hif3α*, *HIF3A*TO cultured in differentiated medium (T0-T7) in presence or absence of cytokines. Relative gene expression data are reported as 2-ΔΔCt method, normalized to housekeeping gene (b-actin and 18S mRNA) *vs.* T0 WT. Data are expressed as means ± SEM (*n* = 3; ***p ≤ 0.001, **p ≤ 0.01, *p ≤ 0.05). **(B)** Western blot analysis of LC3B-II protein in siHif3α adipocytes cultured in differentiation medium in presence of inflammatory cytokines (T7). The ratio of Lc3B-II/Lc3B-I/Gapdh was measured using ImageJ Analysis tool (*n* = 3; ***p ≤ 0.001, **p ≤ 0.01, *p ≤ 0.05 *vs*. control cells).

Reduced lipid catabolism/degradation (lypolysis) can lead to accumulation of lipids (lipogenesis), adipocyte hypertrophy and storage in lipid droplets (LD), which are related to insulin resistance and increased inflammation. Lipoprotein lipase (LPL) catalyses the hydrolysis of triacylglycerol into glycerol and fatty acids (TAG), whose activity is inhibited at a posttranscriptional level by activation of the angiopoietin-like 4 (*Angplt4*). In conditional *Angplt4*KO cells, the browning of WAT adipocytes is evident, increasing fatty acid oxidation (Singh et al., 2018). We analysed in our cellular models the influence of *Hif3α* on *Angplt4* expression. *Angplt4* was induced during WAT differentiation in WT adipocytes (Figure 3A, black bars) and the overexpression of *HIF3A* further increased *Angplt4* mRNA (grey bars). Cytokines pre-treatment had any significant effect on *Angplt4* at T0 compared to untreated cells and only a mild additional effect during differentiation (T3 and T7), whereas *Angplt4* was nearly absent in si*Hif3α* adipocytes (light grey bars) and the cytokines were unable to rescue its expression, suggesting that the lypogenesis is reduced and, more important, we confirmed a transcriptional control of *Angplt4* by *Hif3α* (Tolonen et al., 2020).

Lipid redistribution seen in si*Hif3α* adipocytes could be suggestive of increased lypolysis by TAG hydrolysis and turnover, requiring autophagic degradation of lipid droplets to produce energy (Kounakis et al., 2019). Then, we evaluated the expression of microtubule associated protein 1 light chain 3 beta (LC3B) in our si*Hif3α* adipocytes (Figure 3B). Western blotting analysis confirmed high levels of LC3-IIB accumulation, further increased by TNF*α*, EGF and VEGF in absence of *Hif3α*. The variation in lipid droplets, increased *Ucp1* expression and lypolysis during differentiation is suggestive of adipocytes with enhanced energy metabolism.

### Hif3α Silencing Increases Mitochondrial Respiration

**FIGURE 4:**
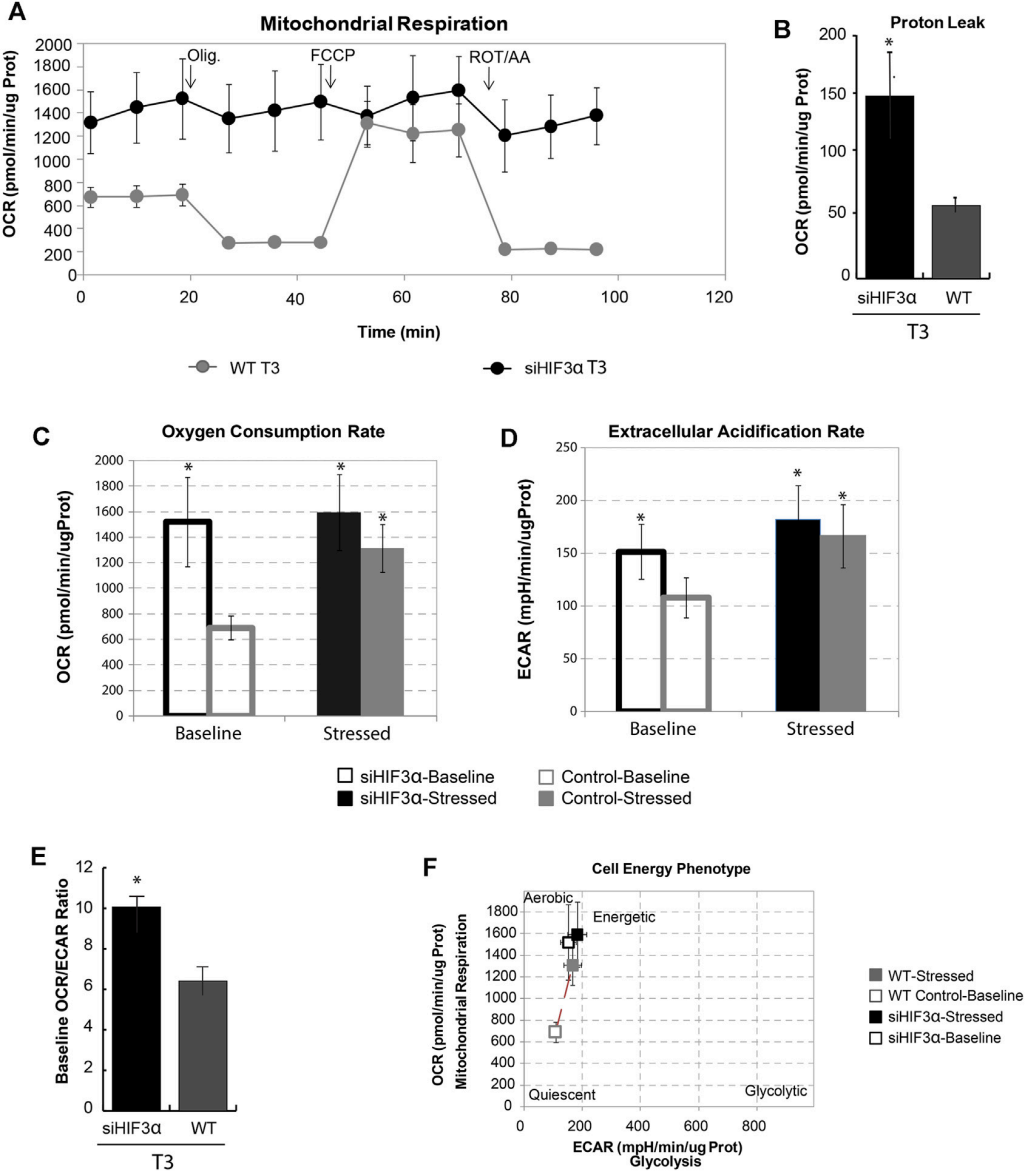
*Hif3α* silencing increases mitochondrial respiration. 3T3-L1 cells were transfected with si*Hif3α* and induced to differentiate **(A)** Seahorse analysis showed the Oxygen consumption rate (OCR) expressed in ug protein. **(B)** Proton Leak. **(C)** Oxygen Consumption Rate. **(D)** Extracellular Acidification Rate. **(E)** Baseline OCR/ECAR ratio **(F)** Mitochondrial respiration *vs.* glycolysis. Values are mean ± *SD* (*n* = 3; *p < 0.05 *vs*. WT or control cells).

The brown/beige and white adipocytes derive from common progenitors, but only the brown/beige ones dissipate stored energy through thermogenesis (Rabiee, 2020). Increased mitochondrial uncoupling or mass could support the link between WAT “browning” and thermogenesis. To do this, we measured the oxygen consumption rate at baseline and after the sequential addition of differential mitochondrial inhibitors in si*Hif3α*-adipocytes, using Seahorse Real-Time Cell Metabolic Analysis (Figure 4A). Silencing of *Hif3α* increased the oxygen consumption rate (OCR) and proton leak compared to WT, showing increased mitochondrial uncoupling, consistent with the characteristics of brown/beige adipocytes (Figures 4A,B). Evaluating the OCR and the extracellular acidification rate (ECAR) in basal and stressed condition, the si*Hif3α* adipocytes were insensitive to the respiratory stress (Figure 4C). We analysed also ECAR, a robust indicator of glycolysis. When highly aerobic cells are stressed, CO_2_ production from the mitochondria can contribute to ECAR and over-report the contribution of glycolysis to metabolic potential. Silencing of *Hif3α* increased ECAR, both in basal and stressed conditions (Figure 4D), suggesting an accumulation of cytoplasmic lactate or increased CO_2_ production. The extracellular acidification when a glucose molecule is oxidized to CO_2_ is three times greater when it is converted to lactate. We calculated baseline OCR/ECAR ratio as a marker of susceptibility to background CO_2_ effect (SE OCR/ECAR >4). *De facto*, the baseline OCR/ECAR ratio indicated that the stressed ECAR parameter including both glycolysis and mitochondrial activity for each cell type was due to the prominent contribution of CO_2_ to ECAR, mainly in si*Hif3α* adipocytes (Figure 4E). Moreover, baseline and stressed phenotype evaluation indicates a large increase in baseline activity (open symbols) and slight increase in utilization of both pathways in response to mitochondrial stressors in si*Hif3α* vs. WT, suggesting a cell energetic phenotype (Figure 4F), not an increase in mitochondria mass evaluated by MitoTracker staining (data not shown).

**FIGURE 5:**
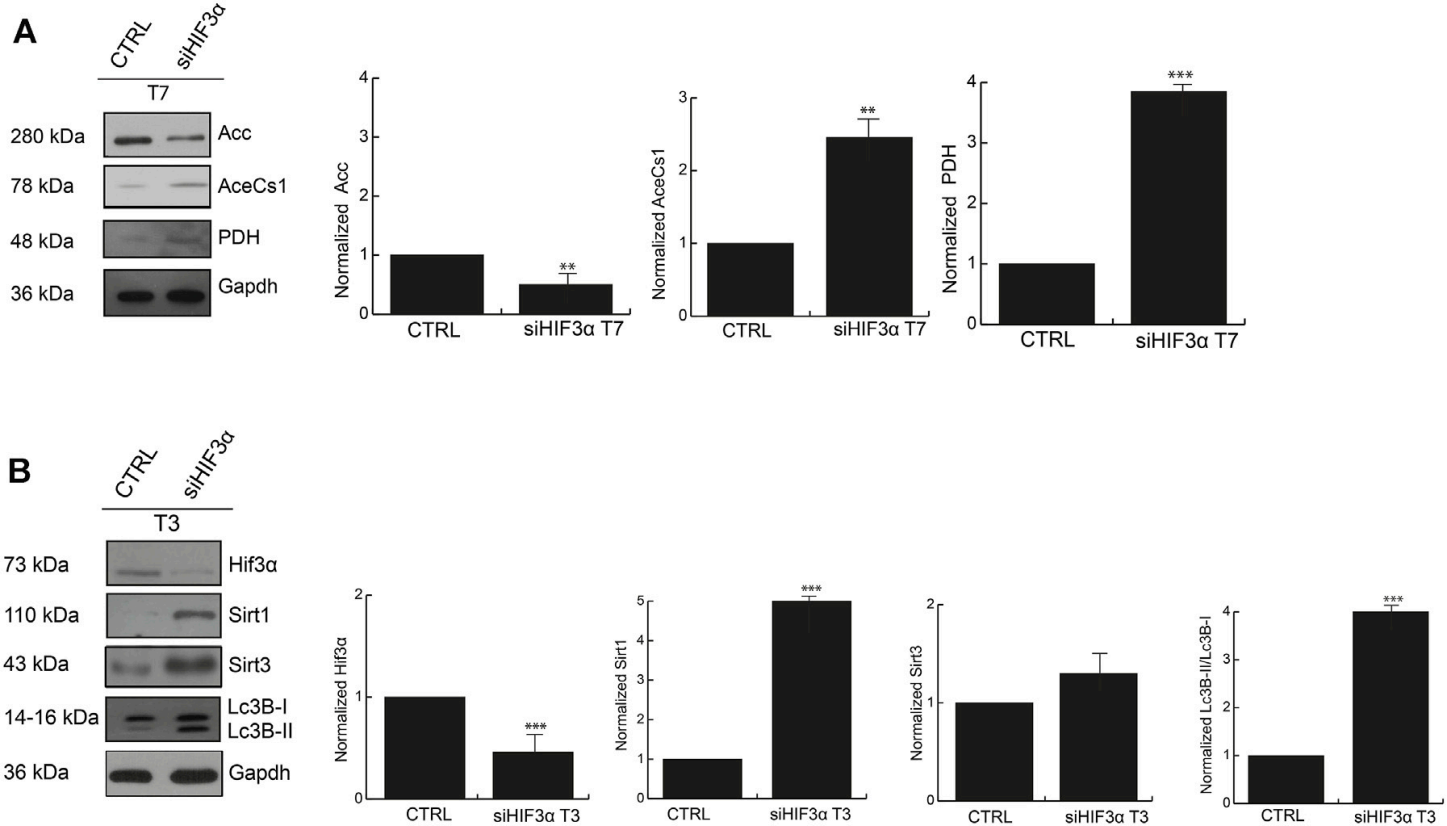
The inhibition of *Hif3α* induces fatty acids synthesis enzymes **(A)** Western blot analysis of Acc, AceCS1 and PDH in si*Hif3α* after 7 days of differentiation induction. SIRTs are activated in absence of *Hif3α* **(B)** Western blot analysis of Hif3α, Sirt1, Sirt3 and Lc3B-I/II detected in si*Hif3α* adipocytes at T3. Densitometry values are mean ± SEM of biological triplicates and are graphed as normalized for relative housekeeping (Actin or Gapdh) using the ImageJ Gel Analysis tool and expressed as fold change. Gapdh was showed as representative housekeeping. (*n* = 3; ***p ≤ 0.001, **p ≤ 0.01 *vs*. control cells).

The large amount of energy activated in absence of *Hif3α* could derive from increased fatty acid metabolism, thus we investigated the protein levels of pyruvate dehydrogenase (PDH) (pyruvate to acetyl-CoA), acetyl-CoA synthetase (AceCs1) (acetate to acetyl-CoA) and acetyl-CoA Carboxylase (ACC) (Acetyl-CoA to malonyl-CoA) that provide acetyl-CoA for catabolic (TCA cycle), and/or anabolic (fatty acid and cholesterol synthesis) purposes. Western blotting showed that the adipocytes in absence of *Hif3α* (si*Hif3α* adipocytes) showed an increase of AceCS1 and PDH enzymes and downregulation of ACC (Figure 5A). Thus, in absence of *Hif3α*, the enzymes involved in *de novo* fatty acids synthesis are induced, clearly explaining the increment of ECAR (more pyruvate to acetyl-CoA conversion, less pyruvate to lactic acid conversion).

### Hif3α Inhibition Induces SIRT Expression

Acetyl-CoA controls key cellular processes, including energy production, lipid metabolism and autophagy, directly or *via* the epigenetic regulation of gene expression (Wellen et al., 2009). BAT remodelling is mediated by *Pparg* deacetylation and thus activation of SIRT enzymes (Qiang et al., 2012). Then, we hypothesized that increased energetic metabolism and acetyl-CoA accumulation could be linked to SIRT activation, especially SIRT1 and SIRT3, regulated by the bioenergetics status of the cell (Tang, 2016). Thus, we measured Sirt1 and Sirt3 protein levels by WB in si*Hif3α* adipocytes and we found a significant increase of Sirt1 and Sirt3 proteins in silenced adipocytes starting from the day 3 of differentiation, coupled with Lc3-IIB accumulation (Figure 5B).

### The Causal *Hif3A/Ucp1* Relation in Thermogenesis-Induced Model

**FIGURE 6:**
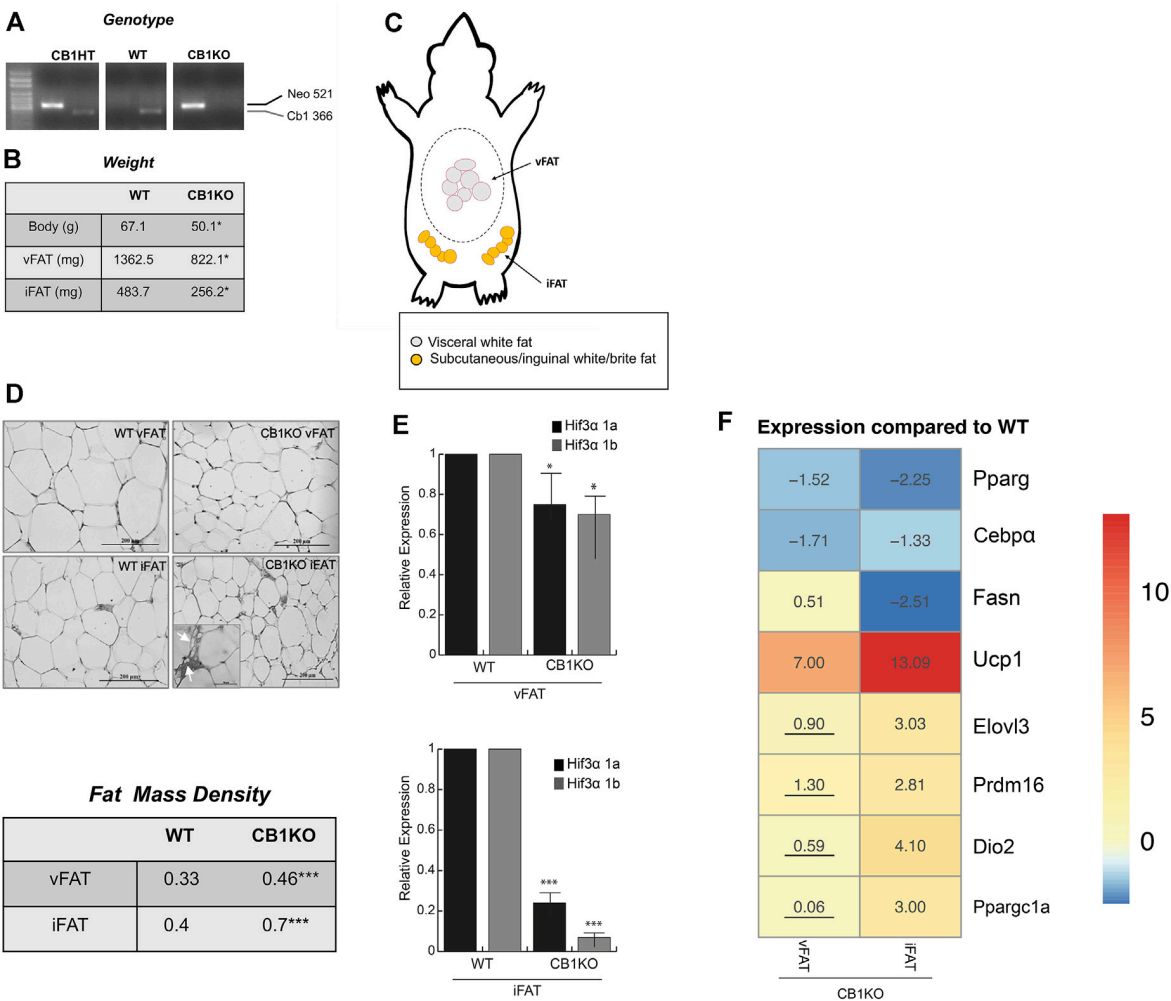
The causal *Hif3α/Ucp1* relation in thermogenesis-induced model. **(A)** Animal genotyping by PCR analysis of genomic DNA. **(B)** Animal and fat depots weights **(C)** Schematic representation of the distribution of adipose tissue depots collected: visceral white fat (vFAT) and inguinal/subcutaneous white fat (iFAT). **(D)** Representative haematoxylin and eosin-stained of vFAT and iFAT sections (upper panel) and fat cell density (lower panel). **(E)** Expression levels of *Hif3α* isoforms gene by qPCR in vFAT and iFAT of WT and CB1KO mice. Relative gene expression data are reported as 2-ΔΔCt, normalized to housekeeping genes (b-actin and 18S mRNA). Data are expressed as means ± SEM (*n* = 3 animals/genotype, ***p ≤ 0.001, *p ≤ 0.05 vs. WT mouse). **(F)** Heatmap represents expression profile of WAT-specific (*Pparg, C/ebpα, Fasn*) and BAT-specific genes (*Ucp1, Elovl3, Prdm16, Dio2, Ppargc1a*) in vFAT and iFAT differentially expressed in CB1KO compared to WT. Values represent the means of the fold change *vs.* WT (*n* = 3 animals/genotype). All values are significant (*p* < 0.05), except those underlined.

The endocannabinoid system plays a role in energy metabolism and CB1-deficient mice as well as adipocyte-specific deletion of CB1 showed decreased body weight and adiposity, with the appearance of brown-like adipose tissue in fat depots, increased fat cell density and enhanced energy expenditure (Wagner et al., 2011; Ruiz de Azua et al., 2017). Hence, this model served as an ideal model to study the relation between *Hif3α* and *Ucp1*. Once identified the WT and KO mice (Figure 6A), we measured the body weight of WT vs. CB1KO animals and, as expected, CB1KO mice showed decreased body weight (Figure 6B). We isolated visceral and inguinal adipose depots (vFAT and iFAT) (Figure 6C) and we confirmed an interesting reduction in mg of the fat tissues in CB1KO mice (Figure 6B). The histological observation of the vFAT and iFAT showed a significant morphological modification: CB1KO mice displayed the presence of adipocytes with reduced cell size and increased cell density in iFAT compared to WT (Figure 6D upper panel). The count of adipocytes per area (adipocyte number/100 mm^2^) indicated that adipocyte density was significantly higher (***p < 0.001) in CB1KO, both in vFAT and iFAT compared with WT, demonstrating an effect of CB1KO on fat cell density (Figure 6D lower panel). Interestingly, an increase of multilocular droplets as in brown-like adipocytes in iFAT was detected (indicated by arrow).

Then, we measured the expression level of *Hif3α* isoforms in both fat depots of WT and KO mice (vFAT and iFAT) (Figure 6E). *Hif3α* mRNAs were expressed in fat tissues of WT mice, whereas a significant reduction of Hif3α mRNA levels was displayed in vFAT, mainly in iFAT, of CB1KO mice. We investigated the expression levels of specific key regulators of adipogenesis and we found in iFAT of CB1KO mice an increase of Ucp1, *Elovl3, Prdm16, Dio2, Ppargc1a* and a decrease of *Pparg, C/ebpα, Fasn* mRNAs, compared to WT (Figure 6F), confirming the role of Hif3a in controlling adipocytes metabolism *in vivo*. Reduced level of *Hif3α* paralleled by a consistent increment of Ucp1 suggested the appearance of brown/beige adipocytes in fat depots, confirming the causal *Hif3α/Ucp1* relation also *in vivo*.

## Discussion

Taken together, our results showed that *Hif3α* plays a role in adipogenesis controlling WAT differentiation. The inhibition of *Hif3α* unexpectedly promoted the “browning” of white adipocytes, with an increase in energy metabolism *via Ucp1*-mediated thermogenesis *in vitro* and in thermogenesis-induced model *in vivo*. Thus, *Hif3α* is a regulator of BAT induction, guiding beneficial metabolic reprogramming, dissipating energy as heat thereby improving glucose and lipid metabolism, decreasing the insurgence of metabolic disorders.

The causal mechanisms underlying adiposity and metabolic disorders remain unclear.

We speculated that *Hif3α* might control the adipocyte physiology in a dynamic manner: *Hif3α* senses and drives metabolic changes to adapt to inflammatory status that promotes lipid accumulation; if the stressor is removed, the cellular homeostasis can be rescued, but in chronic conditions it becomes maladaptive and the adipocytes start to accumulate lipids, expanding WAT.

Several studies showed that *HIF3A* is methylated and this modification is significantly associated to obesity (Dick et al., 2014). This raises several questions: is *HIF3A* methylation a consequence of increased adiposity? Or increased meth*HIF3A* is a causal step on the way to obesity?

In response to excess caloric intake, adipose tissue can increase cell size (hypertrophy) and/or cell number (hyperplasia) (Choe et al., 2016). In humans, fat cells undergo dynamic turnover and, more important, adipocyte number is determined during childhood and adolescence, and maintained in adults even after important weight loss (Spalding et al., 2008; Nishtar et al., 2016). Therefore, abnormal BMI during the adolescence predisposes to obesity, suggesting an epigenetic memory. Interestingly, Pan et al. (2015) show that *in utero* meth*HIF3A* correlated with high BMI in adults. Gestational diabetes mellitus (GDM) increases risk of obesity and diabetes. Antoun et al. (2020) demonstrates that life style intervention in dysglycemic pregnant mothers modifies infant’s epigenomes; *HIF3A* remained methylated in hyperglycemic mothers, whereas is active in normoglycemic ones. Methylation of *HIF3A* locus is also modifiable by diet. Huang et al. found that vitamin B and folate intake influences the methylation of a specific SNP in *HIF3A* locus (Huang et al., 2015), indicating that deprivation of these oligoelements would predispose to obesity by modifying the epigenome (Boachie et al., 2020). In line with this evidence, Petrus et al. (2020) show that glutamine is markedly reduced in obese patients and reduced levels modify O-GlcNAcylation of promoters of inflammatory genes, activating them. Administration of glutamine in high fat diet-fed mice decreases inflammation, enhances adipocytes metabolism remodelling the chromatin *via* different mechanisms (Petrus et al., 2020).

In other cells of the body, the balance between metabolites and chromatin remodelling coexist as in immune cells, in which malonyl-CoA for histone malonylation (Li et al., 2018), lactate for histone lactylation (Zhang et al., 2019) modifies epigenetically different transcriptional programs according to specific metabolic state.

Therefore, obesity can be considered an epigenetic disorder, in which DNA modifications (methylation, acetylation, etc.) are the mechanism by which the genome of adipocytes can be modified, predisposing to later disease risk (Hanson and Gluckman, 2014). Genome-wide association studies of human fat lypolysis identify *HIF3A* expression in mature and precursor adipocytes and knock-down of *HIF3A* in human MSCs increased fatty acid oxidation, suggesting that reported studies and our results support the hypothesis that *HIF3A* is relevant in adipogenesis and methylation is secondary to disrupt the metabolism leading to obesity.

To note, epigenetic deregulation is also a hallmark of cancer initiation and progression and it is now clear that obesity increases the risk to develop a cancer. As speculation, we looked at the expression data of *HIF3A* by Gene Expression Profiling Interactive Analysis (GEPIA) in cancers mostly associated to obesity (breast, oesophageal, pancreatic, stomach, thyroid, and uterine cancers): *HIF3A* is significantly downregulated in tumor *vs.* normal tissue and the promoter regions are methylated (Supplementary Figure S3A), suggesting a causal correlation between methylation of HIF3a in obese patients that subsequently develop cancer. Furthermore, the malignant cells rewire their metabolism to support cancer metabolism and growth.

Future studies are needed to understand the dynamics of this metabolic and epigenetic crosstalk, but this report provides evidence that *HIF3A* is a strong candidate gene with novel regulatory function in fat metabolism and can pave the way for developing metabolic therapy to target the abnormal energy intake in adipocyte cells, enhancing human health.

## Data Availability

The raw data supporting the conclusion of this article will be made available by the authors, without undue reservation.
