# Supplementary figures and images for: HIF3A Inhibition Triggers Browning of White Adipocytes via Metabolic Rewiring
Source: Front Cell Dev Biol. 2022 Jan 12;9:740203. doi: 10.3389/fcell.2021.740203 (PMC8790297; doi:10.3389/fcell.2021.740203)

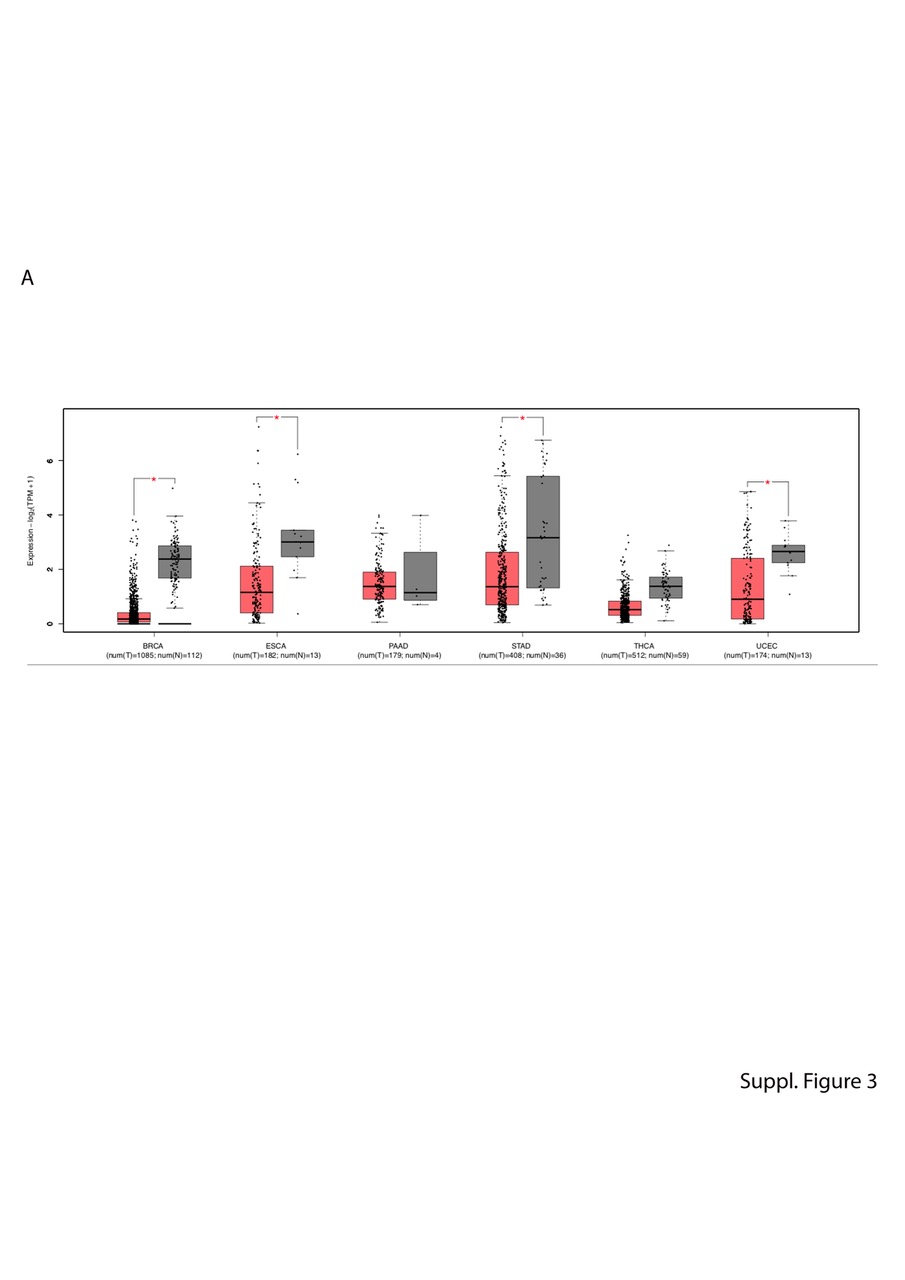

Supplement: Supplementary file 1 [file Image3.JPEG]

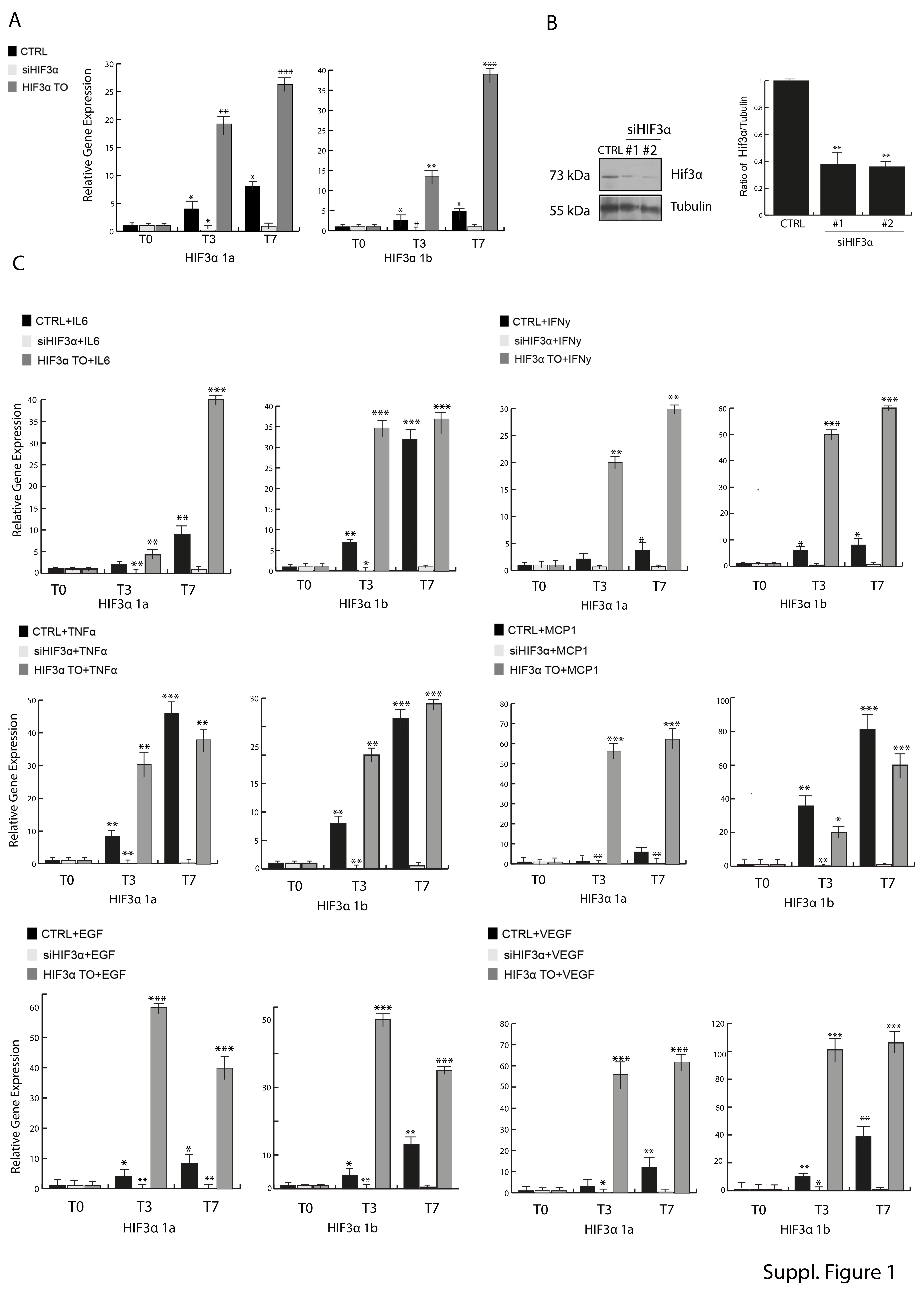

Supplement: Supplementary file 2 [file Image1.JPEG]

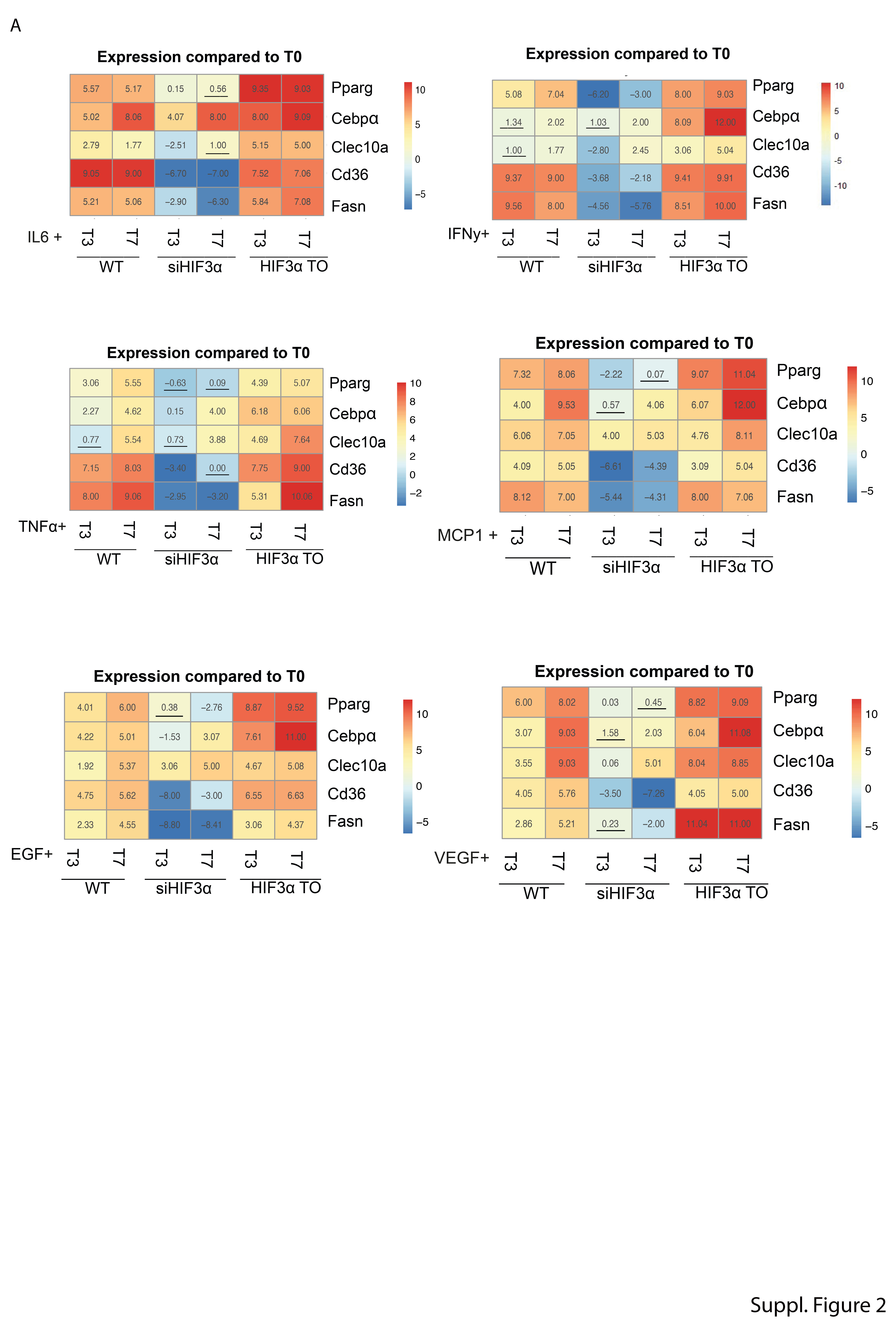

Supplement: Supplementary file 3 [file Image2.JPEG]
