# Supplementary material for: HIF3A Inhibition Triggers Browning of White Adipocytes via Metabolic Rewiring
Source: Front Cell Dev Biol. 2022 Jan 12;9:740203. doi: 10.3389/fcell.2021.740203 (PMC8790297; doi:10.3389/fcell.2021.740203)
Supplement: Supplementary file 4 [file DataSheet1.docx]

Supplementary Material

**Supplementary Figure 1**: ***Hif3α* expression in WT, si*Hif3α,* and *HIF3A*TO (A)** Expression levels of *Hif3α* isoforms by qRT-PCR in WT, si*Hif3α*, *HIF3A*TO adipocytes during differentiation (T3 and T7). Relative gene expression data are reported as 2-ΔΔCt method, normalized to housekeeping gene (b-actin and 18S mRNA). Data are expressed as means ± SEM *vs*. T0WT; (n=3; ****p* ≤ 0.001, ***p* ≤ 0.01, **p* ≤ 0.05). **(B)** *Hif3α* gene was silenced by targeting all isoforms of *Hif3α* transcript by siRNA transfection. The efficiency of gene silencing was checked by immunoblotting. Densitometry values are the mean ± SEM normalized for Tubulin using the Image J Gel Analysis tool, and expressed as fold change; (n = 3; ***p* ≤ 0.01 vs. control cells). (**C**) Expression levels of *Hif3α* isoforms by qPCR in WT, si*Hif3α*, *HIF3A*TO cultured in differentiated medium in presence of pro-inflammatory cytokines (T3 and T7). Data are expressed as means ± SEM *vs.* T0WT; (n=3; ****p* ≤ 0.001, ***p* ≤ 0.01, **p* ≤ 0.05).

**Supplementary Figure 2: Pro-inflammatory cytokines enhance expression of WAT marker genes in presence of *Hif3α***. **(A)** Analysis of the main WAT genes (*Pparg, C/ebpα, Clec10a, Cd36, Fasn*) in WT, si*Hif3α*, *HIF3A*TO cultured in differentiated medium (T3 and T7) in presence of inflammatory cytokines. Heatmap represents expression profile of WAT genes differentially expressed across the different experimental conditions. Values represent the means of the fold change *vs.* T0WT (n=3). T0WT is calculated as 1, thus >1 means upregulation, <1 means downregulation. All values are significant (p ≤0.01) except those underlined.

**Supplementary Figure 3: (A)** Gene Expression Profiling Interactive Analysis (GEPIA) of HIF3A in tumor vs normal tissue.

**
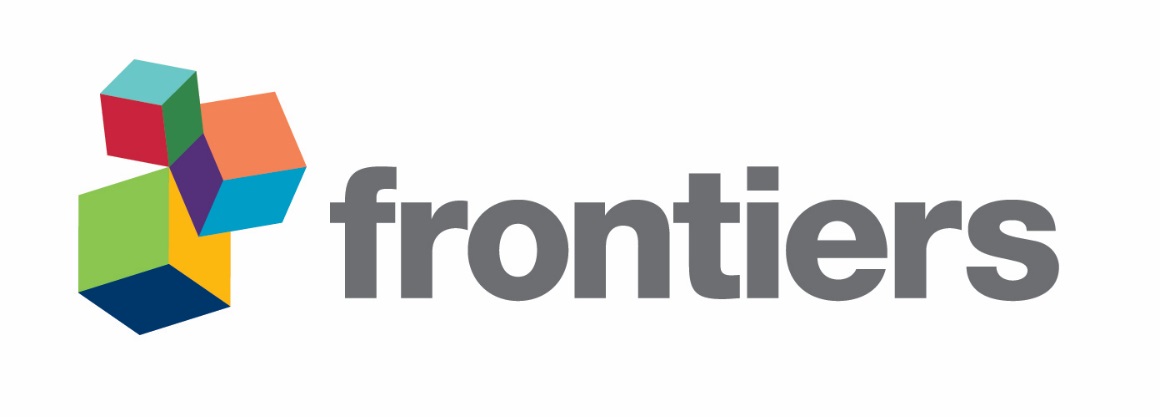
**
